# Including diffusion time dependence in the extra-axonal space improves in vivo estimates of axonal diameter and density in human white matter

**DOI:** 10.1016/j.neuroimage.2016.01.047

**Published:** 2016-04-15

**Authors:** Silvia De Santis, Derek K. Jones, Alard Roebroeck

**Affiliations:** a CUBRIC, School of Psychology, Cardiff University, Cardiff CF10 3AT, UK; b Faculty of Psychology & Neuroscience, Maastricht University, Maastricht, The Netherlands; c Neuroscience & Mental Health Research Institute, Cardiff University, CF10 3AT, UK

**Keywords:** CHARMED, White matter microstructure, STEAM diffusion MRI, Diffusion time, Axonal diameters, Axonal density

## Abstract

Axonal density and diameter are two fundamental properties of brain white matter. Recently, advanced diffusion MRI techniques have made these two parameters accessible in vivo. However, the techniques available to estimate such parameters are still under development. For example, current methods to map axonal diameters capture relative trends over different structures, but consistently over-estimate absolute diameters. Axonal density estimates are more accessible experimentally, but different modeling approaches exist and the impact of the experimental parameters has not been thoroughly quantified, potentially leading to incompatibility of results obtained in different studies using different techniques. Here, we characterise the impact of diffusion time on axonal density and diameter estimates using Monte Carlo simulations and STEAM diffusion MRI at 7 T on 9 healthy volunteers. We show that axonal density and diameter estimates strongly depend on diffusion time, with diameters almost invariably overestimated and density both over and underestimated for some commonly used models. Crucially, we also demonstrate that these biases are reduced when the model accounts for diffusion time dependency in the extra-axonal space. For axonal density estimates, both upward and downward bias in different situations are removed by modeling extra-axonal time-dependence, showing increased accuracy in these estimates. For axonal diameter estimates, we report increased accuracy in ground truth simulations and axonal diameter estimates decreased away from high values given by earlier models and towards known values in the human corpus callosum when modeling extra-axonal time-dependence. Axonal diameter feasibility under both advanced and clinical settings is discussed in the light of the proposed advances.

## Introduction

The possibility of mapping in vivo white matter properties such as the axonal density and diameter has a huge appeal for neuroscientists and clinicians alike. Indeed, changes in axonal morphology have been associated with many conditions of interest. For instance, alterations of axonal diameters have been observed in psychiatric conditions like autism (Piven et al., 1997, Hughes, 2007), dyslexia (Njiokiktjien et al., 1994) and schizophrenia (Randall, 1983, Rice and Barone, 2000). Axon diameters were found to change following exposure to alcohol (Livy and Elberger, 2008), and increase in diameter of the axonal initial segment was reported as an early change in amyotrophic lateral sclerosis (Sasaki and Maruyama, 1992). Axonal damage in the spinal cord of multiple sclerosis patients is associated with both lower densities and higher diameters (Bergers et al., 2002), and axon degeneration precedes cell body death in many neurodegenerative disorders like Huntington's disease (Marangoni et al., 2014).

While diffusion tensor MRI (DT-MRI) has proven to be an incredibly powerful tool for studying white matter properties over two decades (Basser et al., 1994, Basser, 1995), several studies have highlighted its intrinsic lack of specificity to different sub-compartments of cerebral tissue (Pierpaoli et al., 1996, Beaulieu, 2002, Budde and Annese, 2013, De Santis et al., 2014). Understanding the role of white matter (WM) microstructure in brain function, in health and disease demands more specific indices that tap into its sub-components. In this context, multi-compartment modeling approaches based on biophysical models of brain tissue aim to increase the specificity of diffusion MRI analysis, furnishing compartment-specific information. These methods generally require the diffusion signal to be acquired over multiple diffusion weightings, and are thus generically referred to as multi-shell techniques.

For example, the Composite Hindered And Restricted ModEl of Diffusion, or CHARMED (Assaf et al., 2004, Assaf and Basser, 2005), models the signal as the contribution of two different pools: a hindered extra-axonal compartment and one or more intra-axonal compartments, whose properties are characterised by a model of restricted diffusion perpendicular to the fiber axis within impermeable cylinders (Neuman, 1974). A slightly different model called NODDI, which also accounts for the dispersion of axons around the main orientation, was introduced more recently (Zhang et al., 2012). These models are able to estimate the *T*_2_-weighted fraction of water belonging to the intra-axonal pool(s), also interpreted as the axonal density.^1^

The CHARMED framework has been extended to account for different axonal diameters, providing the opportunity to map non-invasively the distribution of axonal calibers within the brain using Axcaliber (Assaf et al., 2008, Barazany et al., 2009) or ActiveAx (Alexander et al., 2010) frameworks. To make the diffusion signal sensitive to axonal diameters, a multi-shell diffusion protocol is acquired over a range of diffusion times. This is achieved by varying the acquisition parameter Δ, which is the time elapsed between the beginning of the two gradient pulses. Varying the diffusion time provides the necessary contrast for estimating the axonal diameter. For a comprehensive review on axonal diameter estimation approaches, see Nilsson et al. (2013).

Axonal diameter methods capture the relative trends for the diameter distribution, returning, for example, higher values in the body of the corpus callosum and lower in the genu and splenium in agreement with histological measures (Aboitiz et al., 1992). However, the absolute diameter value is generally overestimated (Alexander et al., 2010). Histology has extensively reported axonal diameters to lie in the range 0.5–2 μm for human WM (Aboitiz et al., 1992, Tang et al., 1997), while MRI-derived axonal diameter maps in the literature report axonal diameters in the range 5–15 μm (Alexander et al., 2010). This is only partially explained by the fact that larger axons contribute quadratically more signal to the diffusion MR decay (Alexander et al., 2010). The feasibility of obtaining accurate in vivo estimates of axonal diameters in humans on commonly-available hardware has recently been called into question (Burcaw et al., 2015), due to the exceedingly small differences in signal attenuation that must be detected in order to differentiate between different diameters. In addition, Innocenti et al. (2015) pointed out that fibers larger than 3 μm make up no more than 1% of the total fibers.

In the most widely employed diffusion MR sequences (such as those used for DT-MRI in clinical research studies), the extra-axonal compartment indeed represents by far the largest contribution to the signal decay, whereas multi-compartment models focus their attention on the intra-axonal compartment, that contains information on axonal density and diameter. The diffusion in the extra-axonal compartment is typically modeled as a tensor, i.e., it is assumed that it undergoes Gaussian diffusion and, importantly, that the tensor parameters do not depend on diffusion time. Recently, however, it was shown (Burcaw et al., 2013, Novikov et al., 2014) that the extra-axonal compartment diffusion orthogonal to the main fiber orientation is strongly dependent on time, creating a potential source of bias through unmodeled effects in axonal diameter and density estimates (Novikov et al., 2014). Furthermore, it has been shown that this time dependency is a much more pronounced effect than the signal attenuation due to finite inner axonal diameters (Burcaw et al., 2015).

The aims of this work are threefold: 1) to investigate the influence of diffusion time in vivo over a wide range in multi-compartment models using diffusion weighted STimulated Echo Acquisition Mode (STEAM) MR imaging; 2) to study the feasibility of human axonal diameter mapping techniques at 7 T; and 3) to modify current models to estimate axonal diameter distributions and density to account for diffusion time effects in the extra-axonal compartment, with the ultimate goal of obtaining more accurate estimates of axonal density and diameters in vivo in the human brain.

## Methods

### Theory

All multi-compartment frameworks for estimating axonal density and diameter are based on decomposing the signal into (at least) two distinct pools: the extra-axonal compartment, whose diffusion is considered hindered, and the intra-axonal compartment, whose diffusion is considered restricted (Assaf et al., 2004). For a generic orientation **n**, and considering a diffusion weighted STEAM acquisition, this can be formalised as:

(1) $$S\left(q,\Delta \right)=e^{-\mathrm{TM}\left(\Delta \right)/T_{1}}\cdot S\left(0\right)\left[f_{h}*S_{h}\left(q,\Delta \right)+f_{r}*S_{r}\left(q,\Delta \right)\right]$$

where TM is the mixing time, i.e. the time between the two 90^°^ pulses, *T*_1_ is the longitudinal relaxation time, **q** is the reciprocal wavevector, defined as *γδg*/2*π* ⋅ **n** (where *γ* is the gyromagnetic ratio, *δ* is the diffusion gradient duration, *g* is the diffusion gradient amplitude), Δ is the diffusion gradient spacing, *f*_*h*_ and *f*_*r*_ are the signal fraction of the extra-axonal and intra-axonal compartments, respectively, which sum up to one, so that *f*_*h*_ = 1 − *f*_*r*_. Some multi-compartment frameworks also account for an isotropic compartment corresponding to the cerebrospinal fluid, which undergoes free diffusion (Alexander et al., 2010). *T*_1_ decay needs to be taken into account when performing experiments at different diffusion times with a STEAM acquisition, as this implies using different *TM*s.

When considering 3-dimensional space in presence of cylindrical restrictions, the statistical independence of the net displacement distribution implies that the signal can be decomposed as the product of signal perpendicular and parallel to the cylinder's main orientation (Assaf et al., 2004), for both *S*_*h*_ and *S*_*r*_.

The signal from the intra-axonal pool parallel to the fiber orientation undergoes free diffusion according to:

(2) $$S_{r,∥}\left(q_{∥},\Delta \right)=e^{-4\pi^{2}|q_{∥}|{}^{2}\left(\Delta -\delta /3\right)D_{r,∥}}$$

where *q*_∥_ is the projection of the reciprocal wavevector in the orientation parallel to the direction of largest diffusivity and *D*_*r* , ∥_ is the intra-axonal diffusivity parallel to the direction of largest diffusivity.

The signal from the intra-axonal pool orthogonal to the fiber orientation can be described according to several models of restriction within cylinders (Neuman, 1974, Callaghan et al., 1992, van Gelderen et al., 1994), depending on the specific pulse sequence. For the purpose of this study, Van Gelderen's model was used (van Gelderen et al., 1994):

(3) $$S_{r,⊥}\left(q_{⊥},\Delta ,R\right)=e^{-8\pi^{2}{\left|q_{⊥}\right|}^{2}\sum_{m=1}^{\infty }\frac{2D_{f}\alpha_{m}^{2}\delta -2+2e^{-D_{f}\alpha_{m}^{2}\delta }+2e^{-D_{f}\alpha_{m}^{2}\Delta }-e^{-D_{f}\alpha_{m}^{2}\left(\Delta -\delta \right)}-e^{-D_{f}\alpha_{m}^{2}\left(\Delta +\delta \right)}}{\delta^{2}D_{f}^{2}\alpha_{m}^{6}\left(R^{2}\alpha_{m}^{2}-1\right)}}$$

where *D*_*f*_ is the free diffusion coefficient, *R* is the radius of the cylinder and *α*_*m*_ are the roots of the equation *J*'_1_(*α*_*m*_*R*) = 0. *J*'_1_ is the derivative of the Bessel function of the first kind, order 1. The total decay in the restricted compartment can be then written as:

(4) $$S_{r}\left(q,\Delta \right)=\sum_{i}w_{i}\left[S_{r,⊥}\left(q_{⊥},\Delta ,r_{i}\right)\cdot S_{r,∥}\left(q_{∥},\Delta \right)\right]$$

where *w*_*i*_ are defined as:

(5) $$w_{i}=\frac{P\left(r_{i}\right)\cdot A\left(r_{i}\right)}{\sum_{i}P\left(r_{i}\right)\cdot A\left(r_{i}\right)}$$

*P*(*r*_*i*_) is the value of the distribution of each radius *r*_*i*_ and *A*(*r*_*i*_) is the area of the corresponding cylinder, so that *w*_*i*_ are the weights of the in-plane signal attenuation caused by the water spins diffusing in cylinders with radius *r*_*i*_. The weights are normalised to sum up to one and are dimensionless. All models of restriction depend on the size of the restricting boundary *r*_*i*_, which in the case of white matter means that they depend on the axonal diameter (i.e., twice *r*_*i*_). For the purpose of estimating the axonal density, *f*_*r*_, the distribution of axonal radii can be assumed from histological findings, as in the CHARMED model (Assaf et al., 2004, Assaf and Basser, 2005). *f*_*r*_ can then be estimated by acquiring *S*(*q*, Δ) for different *q*. Alternatively, a simpler model of restriction can be used, that assumes zero orthogonal diffusivity in the restricted pool and thus is independent of the size of the restricting geometries, as in the NODDI model (Zhang et al., 2012). If the purpose is instead to estimate the axonal diameter, *S*(*q*, Δ) should be acquired for different *q*-Δ combinations and the scalar diameter index *R* estimated directly from the data, as done in the AxCaliber (Assaf et al., 2008) and ActiveAx (Alexander et al., 2010) frameworks. The AxCaliber framework fits a distribution of diameters, rather that a single diameter, using a two-parameter gamma distribution (called *P*(*r*_*i*_) in Eq. (5)), characteristic of histological findings (Aboitiz et al., 1992), with *R* estimated as the gamma distribution mean. ActiveAx fits instead a single, average diameter.

Diffusion in the extra-axonal compartment is assumed to be hindered and, thus, this compartment has a 3D Gaussian displacement distribution:

(6) $$S_{h}\left(q,\Delta \right)=e^{-4\pi^{2}\left(\Delta -\delta /3\right)q^{T}D_{h}q}$$

where **D**_*h*_ is the extra-axonal tensor. In coherently oriented white matter, **D**_*h*_ is likely to be highly anisotropic, with the fastest diffusion orientation aligned with the predominant fiber orientation. In the CHARMED/AxCaliber frameworks, there are no priors about **D**_*h*_, which is expressed as a generic tensor with six independent components, and these components are estimated in the fit. When describing a cylindrical symmetry with known orientation like the corpus callosum (as in this paper), only two components are actually needed:

(7) $$D_{h}=\left(\begin{array}{lll} D_{h,∥} & 0 & 0\\ 0 & D_{h,⊥} & 0\\ 0 & 0 & D_{h,⊥} \end{array}\right)$$

In the NODDI/ActiveAx frameworks instead, a smaller number of parameters is fitted, as the orientation of largest diffusivity is linked to the orientation of largest diffusivity in the intra-axonal compartment and the values for the orthogonal diffusivities are derived from the longitudinal diffusivity using the tortuosity approximation (Szafer et al., 1995), i.e.:

(8) $$D_{h,⊥}=D_{h,∥}\cdot \left(1-f_{r}\right)$$

leading to the following expression for the diffusion tensor in the hindered compartment:

(9) $$D_{h}=\left(\begin{array}{lll} D_{h,∥} & 0 & 0\\ 0 & D_{h,∥}\cdot \left(1-f_{r}\right) & 0\\ 0 & 0 & D_{h,∥}\cdot \left(1-f_{r}\right) \end{array}\right)$$

In addition, the extra- and intra-axonal diffusivities parallel to the main orientation (*D*_*r* , ∥_ and *D*_*h* , ∥_) are assumed to be equal, although ongoing work is investigating possible deviations (Novikov et al., 2015, Jelescu et al., 2015, Jelescu et al., 2016).

Recently, Novikov and co-workers showed both theoretically (Novikov et al., 2014, Burcaw et al., 2015) and experimentally (Burcaw et al., 2013) that randomness in fiber arrangement in a bundle crucially affects diffusion in the extra-axonal space, making the diffusion orthogonal to the bundle *D*_*h* , ⊥_ dependent on Δ:

(10) $$D_{h,⊥}\left(\Delta \right)=D_{h,\infty }+A\frac{F\left(\Delta /\delta \right)}{\Delta -\delta /3}$$

where *D*_*h* , ∞_ is the bulk diffusion constant, *A* is a characteristic coefficient and *F*(*x*) can be approximated as $\ln\left(x\right)+\frac{3}{2}$ for *x* ≫ 1 (Burcaw et al., 2015). The authors suggest that this dependency should be included in any quantification scheme for adequate fiber characterisation. They also demonstrated that the coefficient A scales approximately as the square of the correlation length, which in turn is proportional to the outer axonal diameter (which includes the myelin sheath).

Given this dependence on axon diameter, we incorporated the time-dependency represented in Eq. (10) into the framework for estimating the axonal density and diameter, i.e., in the extra-axonal signal decay of Eq. (6). Since, again, statistical independence of displacements parallel and perpendicular to a restrictive barrier leads to a simple product relationship between the diffusion-weighted signals measured along these orthogonal directions (Assaf et al., 2004), the diffusion tensor in Eq. (6) can be written as:

(11) $$D_{h}=\left(\begin{array}{lll} D_{h,∥} & 0 & 0\\ 0 & D_{h,\infty }+A\frac{\ln\left(\Delta /\delta \right)+3/2}{\Delta -\delta /3} & 0\\ 0 & 0 & D_{h,\infty }+A\frac{\ln\left(\Delta /\delta \right)+3/2}{\Delta -\delta /3} \end{array}\right)$$

where *D*_∥_ is the longitudinal diffusivity. This is expected to impact the frameworks for estimating the axonal density that use a tortuosity approximation, and to impact the frameworks to estimate the axonal diameter. Conversely, this is expected not to impact frameworks like CHARMED, where the acquisition is performed for a single, fixed diffusion time and where there is no prior on the orthogonal diffusivity, which is a free parameter in the fit.

### Simulations

We used two different and complementary approaches to investigate the impact of the diffusion time in modeling axonal density and diameter. First, we used simulated signals to investigate the impact on axonal density estimates of neglecting the dependency on the diffusion time of the extra axonal signal, i.e., the scenario in which Eq. (11) is correct but the data are fitted to Eq. (6) instead (as done in current methods). Second, we used Monte Carlo simulations to investigate the sensitivity of axonal diameter estimates under current settings and to characterise the impact on the axonal diameter of neglecting the dependency on the diffusion time of the extra axonal signal. Monte Carlo simulations are needed to model the realistic axonal geometries that are required to mimic axonal diameter distributions.

#### Signal simulations

Signal simulations were generated in Matlab (R2012b, The Mathworks). The diffusion signal was generated for a fiber bundle oriented along the x axis. The simulated STEAM acquisition scheme was the same one used for the in vivo experiments: *δ* = 17 ms, Δ = 48,60,80,100,120,140,160,180,195 ms, 4 b-values (500, 1000, 2000 and 4000 s/mm^2^) for each Δ, by using 1 (at maximum 70 mT/m) or 2 (resulting in a magnitude of $70\times \sqrt{(}2)=99$ mT/m) simultaneous gradients perpendicular to the fiber axis, plus two unweighted images for each Δ. The signal was simulated using Eq. (1), including the dependency on diffusion time as given by Eq.(11), with the following parameters: free diffusion coefficient *D*_*f*_ = 1 ∗ 10^− 3^mm^2^/s and 2 ∗ 10^− 3^mm^2^/s, axonal density *f*_*r*_ = 0.3 and 0.5, axonal radius *R* = 0.25 and 1.5 μm, *D*_*h* , ∞_ = 0.5 ∗ 10^− 3^mm^2^/s, *A* = 2 mm^2^ and relaxation time *T*_1_ = 800 ms. When using stimulated echo at different diffusion times, the spins undergo *T*_1_ relaxation, which further attenuates the signal. 10,000 noisy repetitions were generated adding Rician noise at SNR = 30, to match the SNR of the in vivo data (see next paragraph). The fitting routine was written in Matlab (The Mathworks, Natick, MA), based on nonlinear least-squares estimation. As a preliminary step, the complete model was fit to analyze accuracy in all the variables. Across all the tested configurations, the mean accuracy was larger than 80% for all the variables except A, for which a smaller value of 67% was measured. Then, to perform the comparison between tortuosity-based and non tortuosity based approaches, each diffusion time was considered separately to estimate the axonal density *f*_*r*_ using Eq. (1), which in turn uses Eqs. (2), (3), (4), (6). Eq. (6) was applied with and without fixing the orthogonal diffusivity for the extra-axonal compartment, i.e., using *D*_*h*_ from Eq. (9) or from Eq. (7). Since the data were normalised to the b = 0 images (*S*(0) in Eq. (1)) and a single parameter (*D*_*h* , ∥_) was used to describe the non-restricted diffusivities *D*_*r* , ∥_, *D*_*h* , ∥_ and *D*_*f*_, the only free parameters in the fit were *f*_*r*_, *D*_*h* , ∥_ and *D*_*h* , ⊥_ in the former case, *f*_*r*_ and *D*_*h* , ∥_ in the latter. Please, note that the fiber orientation was not fitted; the ground truth (x-axis) was used instead. As such, for each diffusion time, mean and standard deviation of all parameters were obtained.

#### Monte Carlo simulations

Monte Carlo simulations were generated using Camino (Cook et al., 2006). Different axonal 3D geometries were generated comprising parallel cylinders with gamma-distributed radius. The parameters of the gamma distribution were chosen according to histology reported in (Aboitiz et al., 1992): the histograms of axonal counts along the corpus callosum were digitised and fitted to a gamma distribution (Barazany et al., 2009). As such, 4 different distributions, characterised by different mean axonal diameter ranging from 1.26 to 1.94 μm, were obtained. In addition, 3 different values for the walker's diffusivity, assumed to be the same in the intra- and extra-axonal compartments, were used: *D*_*f*_ = 0.7 ∗ 10^− 3^ mm^2^/s, *D*_*f*_ = 1.5 ∗ 10^− 3^ mm^2^/s and *D*_*f*_ = 2.4 ∗ 10^− 3^ mm/s. The restricted signal fraction was set to 0.6. 10^5^ walkers and 10^4^ timesteps were used for all simulations. The simulated acquisition scheme was the same as the signal simulations (4 b-values, 9 Δs) and reported above. The relaxation time was *T*_1_ = 800 ms. For each configuration, 30 repetitions were generated using different starting points. Rician noise was added to the signal decay at SNR = 25, 40 and 55. As a preliminary step, only low b-value (*b* ≤ 1000 s/mm^2^) images acquired orthogonally to the corpus callosum orientation ([0 1 0] and [0 0 1]) were considered and fitted to a simple exponential decay to measure the DT-MRI diffusion coefficient orthogonal to the fiber orientation, according to:

(12) $$S_{\mathrm{DTI}}\left(b\right)=e^{-bD_{\mathrm{DTI}}}.$$

This was to reproduce and verify the dependency of the diffusion coefficient orthogonal to the axonal bundle on the diffusion time. All data were used to fit the axonal diameter, both using the conventional framework for estimating axonal diameter, that does not account for the dependency in Eq. (10), and also including this dependency. The fitting routine was written in Matlab (The Mathworks, Natick, MA), based on nonlinear least-squares estimation. The first step is to estimate *T*_1_ from the nominal b = 0 images at different STEAM mixing times. The estimated *T*_1_ is then fixed in the axonal diameter fit (Alexander and Dyrby, 2012). The axonal diameter distribution, i.e., the weights in Eq.(4), was modeled as a continuous Poisson distribution (Ilienko, 2013), that has a single parameter to define both the mean and the width. The Poisson distribution has the advantage of reducing the number of fitted parameters, but still accounts for the inhomogeneous composition of fibers (Aboitiz et al., 1992). Data were fitted using Eq.(1), which in turn uses Eqs. (2), (3), (4) and Eq.(6). Eq.(6) was applied with and without accounting for the diffusion time dependency in the extra-axonal compartment, i.e., using *D*_*h*_ from Eq. (9) or from Eq. (11). Since again *D*_*r* , ∥_ and *D*_*f*_ were assumed to be equal to *D*_*h* , ∥_, the fitting parameters were *T*_1_, *D*_∥_, *f*_*r*_, *D*_∞_, A and the mean axonal radius *R* for the proposed approach, *D*_∥_, *f*_*r*_, *D*_⊥_ and the axonal radius *R* for the earlier method without diffusion time dependency. Please, note that the fiber orientation was not fitted; the ground truth (x-axis) was used instead. For each configuration, the percentage of the signal attenuation associated with, respectively, the extra-axonal and the intra-axonal compartments was also calculated.

### Data acquisition

Diffusion acquisition was performed at 7 T with a STEAM sequence which allows for a large range of diffusion times while minimising echo attenuation caused by *T*_2_ relaxation. 9 healthy subjects (mean age: 29 ± 5 years) with no history of neurological diseases participated in the study, after giving their informed written consent. The study was approved by the local ethics committee. The protocol comprised a diffusion-weighted STEAM echo-planar sequence with the following parameters: TE/TR = 67/6200 ms, *δ* = 17 ms, *TM* = 14.4, 26.4, 46.4, 66.4, 86.4, 106.4, 126.4, 146.4, 161.4 ms, corresponding to Δ = 48, 60, 80, 100, 120, 140, 160, 180, and 195 ms, 4 b-values (500, 1000, 2000 and 4000 s/mm^2^) for each Δ, by using 1 (at maximum 70 mT/m) or 2 (resulting in a magnitude of $70\times \sqrt{(}2)=99$ mT/m) simultaneous gradient axes. Data were acquired from 24 slices positioned around the midsaggital corpus callosum. The focus of this study was the corpus callosum, which consists of fibers homogeneously oriented along the left–right (L-R) direction in the midsaggittal plane (arbitrarily denoted as the x-axis [1 0 0]). As such, the sampled diffusion gradients were oriented along 4 perpendicular and 1 parallel directions: [0 1 1], [0–1 1], [0 1 0],[0 0 1] and [1 0 0], plus two unweighted images for each Δ. The total number of collected measurements for each subject was 216, each with 2 averages. An additional HARDI protocol was acquired with a pulsed gradient spin echo EPI sequence to reconstruct the corpus callosum and recover local fiber orientation information, using the following parameters: TE/TR = 57.6/7500 ms, 60 uniformly distributed gradient orientations (Jones et al., 1999), 6 b0, maximum b-value of 2000 s/mm^2^. Acquiring a HARDI protocol to fit the fiber orientations and selecting locations with L–R fiber orientations avoids errors due to mis-alignment of the corpus callosum centre with respect to the L-R direction of the scanner. The resolution of all scans was 2 mm isotropic and the total acquisition time was less than 25 min. The SNR of the highest-Δ b0 image was calculated using the difference method (Murphy et al., 1993) and returned values between 25 and 50 across white matter. To rule out any artifactual dependency on the diffusion time, a spherical phantom filled with oil also underwent the same protocol. To investigate the impact of including smaller Δs, for one of the subjects an additional diffusion protocol was acquired with the following parameters: TE = 48 ms, *δ* = 12 ms, Δ = 38, 60, 80, 100, 120, 140, 160, 180, and 195 ms, 4 shells of b-value 250, 500, 1000 and 2000 s/mm^2^. The other parameters stayed the same. As expected, this lead to a reduction of the achievable TE and a reduction of the maximum applicable b-value.

### Data processing

Motion and distortion corrections were performed using ExploreDTI software (Leemans et al., 2009). ExploreDTI was also used to analyze the HARDI data: whole brain tractography was obtained for each subject in native space using constrained spherical harmonic deconvolution (Tournier et al., 2004). Track termination was based on a fiber orientation density amplitude threshold of 0.1. Waypoints were then defined to virtually dissect the corpus callosum. Axonal density maps were separately reconstructed for each diffusion time, using an in-house fitting routine written in Matlab (The Mathworks, Natick, MA), with and without fixing the orthogonal diffusivity for the extra-axonal compartment. Then, all the data were used to generate maps of the axonal diameter using the modified version of the AxCaliber approach, where the extra-axonal tensor incorporated the dependency on the diffusion time as in Eq. (11). For both axonal density and diameter, the fit was the same as described in the Simulation session; the only difference was that the fiber orientation for each voxel was taken from the HARDI tractography, to minimise errors due to misalignment of the fiber tract with respect to the gradient axes.

### Statistical analysis

To perform a group analysis over all the acquired subjects for both axonal density and axonal diameter, the anterior–posterior central outline of the corpus callosum on the midsaggital plane was extracted in each subject. The central slice was first identified by finding the slice that has the mean principal eigenvector orientation as close as possible to the *x* axis in the corpus callosum. Then, the outline was manually drawn using Matlab and divided into 20 points. At each point, the parameters of interest were estimated using bilinear interpolation. To analyze the influence of the diffusion time on the estimates, a 2-way ANOVA was performed on the profiles using location and diffusion time as factors. To test whether the trend of the axonal density versus the diffusion time was increasing, decreasing or flat, the Bayesian information criterion (Freidlin et al., 2007) was used to choose the function that best fitted to the data. To test the correlation between mean axonal diameter and mean axonal density, linear and quadratic fits were tested using the regression routine in Matlab.

## Results

### Diffusion in the extra-axonal space

**Fig. 1:**
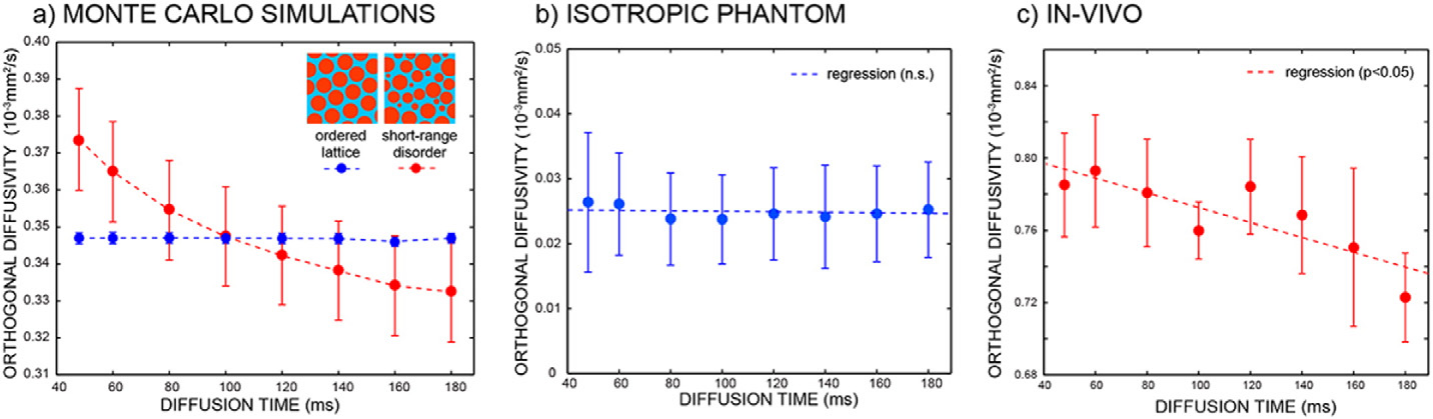
Extra-axonal diffusion coefficient orthogonal to the main fiber orientation as a function of increasing diffusion time for Monte Carlo simulations (a), isotropic phantom (b) and in-vivo data averaged over all the subjects. For the simulations, both ordered (blue) and short-range disordered (red) substrates are used.

Fig. 1 shows the extra-axonal diffusion coefficient orthogonal to the main fiber orientation as a function of increasing diffusion time, for simulated data, for an isotropic phantom and for in-vivo data averaged over the whole corpus callosum and across all subjects, respectively. The dependency on the diffusion time only arises in disordered substrate, as predicted by theory (Novikov et al., 2014) and as shown in Fig. 1a. While the isotropic phantom show a flat trend for increasing diffusion times (Fig. 1b), real data show a decreasing trend, reflecting the complex geometry of white matter.

### Simulations

**Fig. 2:**
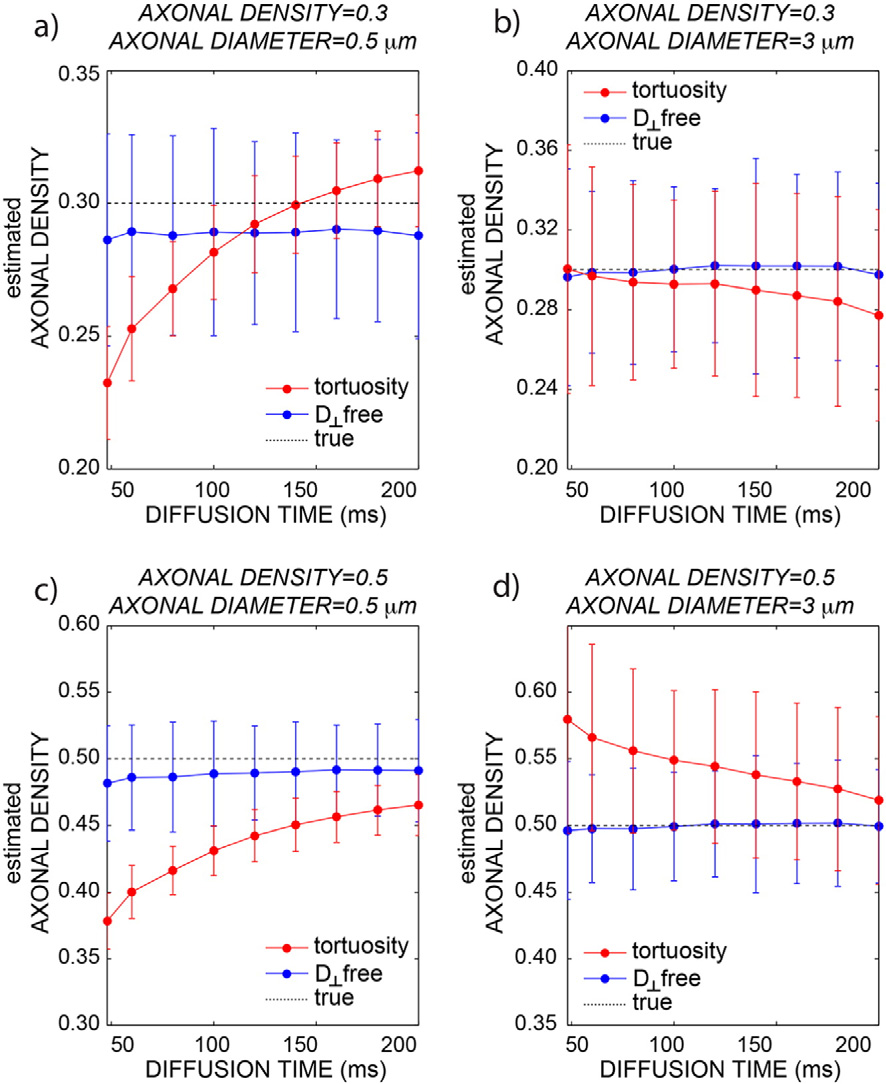
Simulations generated using the signal decay predicted by Novikov et al. (2014), i.e. including the extra-axonal axial diffusion dependency on the diffusion time. The axonal density is plotted as a function of the diffusion time with (red) and without (blue) fixing the orthogonal diffusivity for the extra-axonal compartment. The dotted line is ground truth. Data are reported for different combinations of axonal density and diameter: 0.3/0.5 μm (a), 0.3/3 μm (b), 0.5/0.5 μm (c) and 0.5/3 μm (d).

The effect of disregarding the dependency on diffusion time of the extra axonal signal was evaluated first by simulations. Fig. 2 shows the plot of the axonal density versus diffusion time when the data are analyzed without any prior on the orthogonal extra-axonal diffusivity, i.e., without using the tortuosity approximation (blue line) or using it (red line) for *D*_0_ = 1 ∗ 10^− 3^mm^2^/s. The same plot for *D*_0_ = 2 ∗ 10^− 3^mm^2^/s show a similar trend (data not shown). The simulations are repeated for different axonal diameters (0.5 and 3 μm) and for different axonal densities (0.3 and 0.5), reflecting the variability found in vivo. When the full extra-axonal tensor is fitted (i.e., all six unique elements are estimated), there is a slight tendency towards underestimation but no dependence on diffusion time. When instead the tortuosity approximation is used, there are strong dependencies on diffusion time (both increasing and decreasing) and a complex pattern of interactions of that dependence on axonal diameters and density, leading to both over and underestimation of the axonal density. The precision appears to be higher for the model using the tortuosity approximation. This is likely due to the fact that when the tortuosity approximation is used, the fit has less parameters to estimate.

**Fig. 3:**
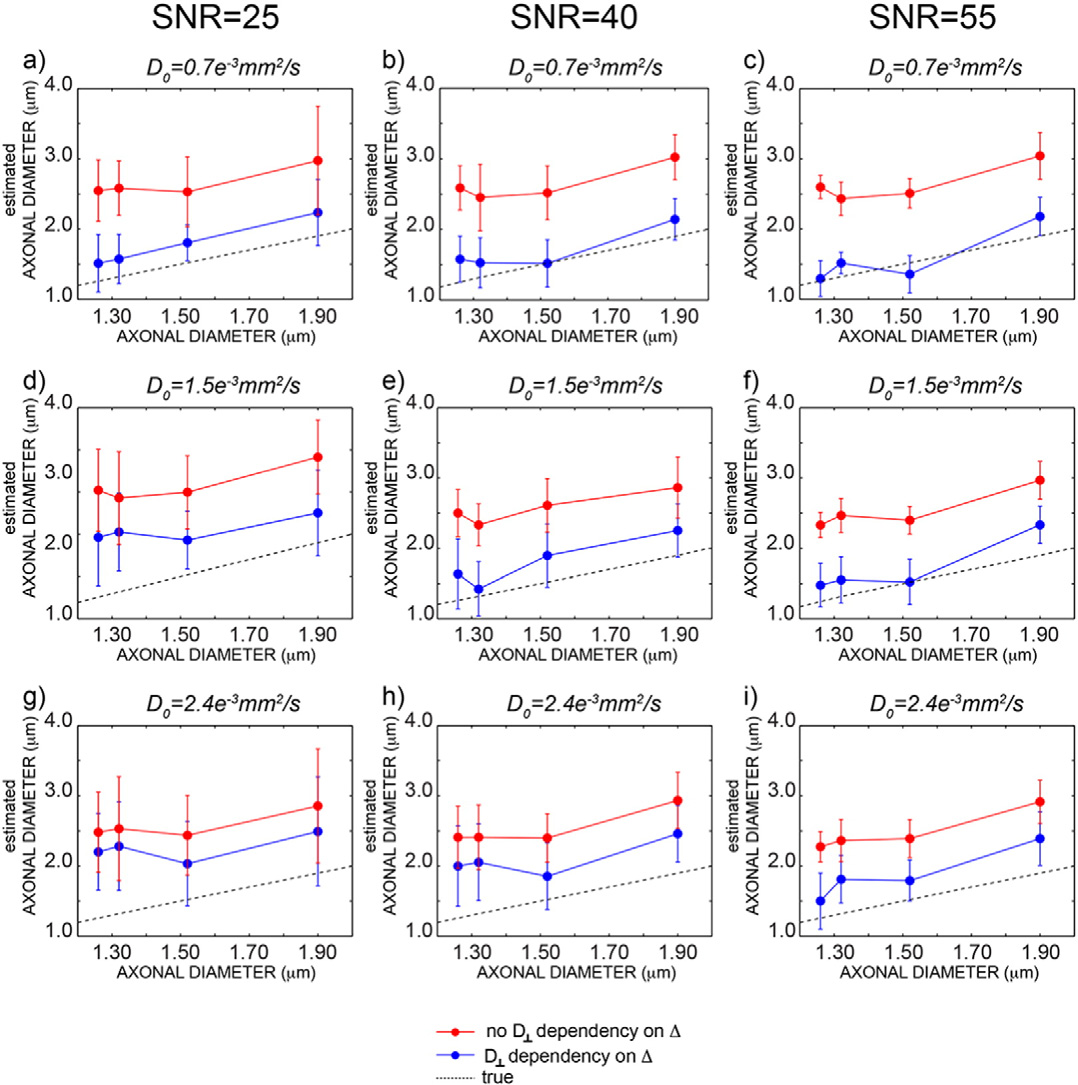
Monte Carlo simulations obtained for different microscopic configurations. True axonal diameter versus estimated axonal diameters including (blue) and not including (red) the delta dependency for *D*_0_ = 0.7 ∗ 10^− 3^ mm^2^/s (a–c), *D*_0_ = 1.5 ∗ 10^− 3^ mm^2^/s (d–f) and *D*_0_ = 2.4 ∗ 10^− 3^ mm^2^/s (g–i). For each value of free diffusivity, the results are reported for SNR = 25,40 and 55 respectively. The dotted line is the line of identity.

**Fig. 4:**
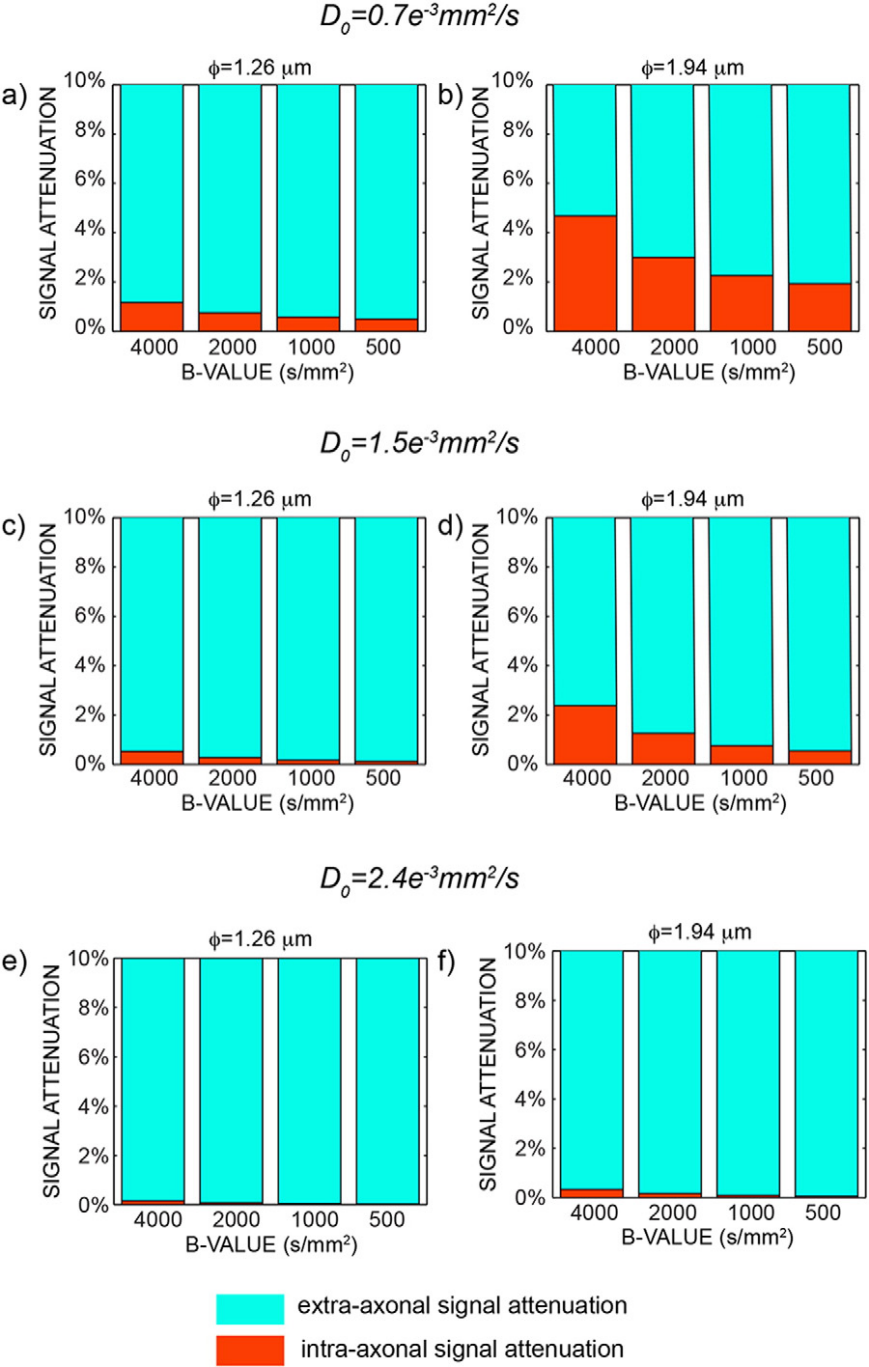
Percentage of extra-axonal (light blue) and intra-axonal (orange) signal attenuation for different b-values and Δ = 48 ms, reported for the smaller (a) and the largest (b) axonal diameters and for *D*_0_ = 0.7 ∗ 10^− 3^ mm^2^/s. c) and d) show the same data for *D*_0_ = 1.5 ∗ 10^− 3^ mm^2^/s, while e) and f) show the same data for *D*_0_ = 2.4 ∗ 10^− 3^ mm^2^/s.

Monte Carlo simulations were used to investigate the sensitivity of axonal diameter measures and to evaluate the impact of the different models on the estimated axonal diameter. Mean and standard deviations of all fitted parameters are reported in Table 1. Fig. 3 shows the estimated axonal diameter using the formula in Eq. (11) (blue) and using the tortuosity model (red) for three different SNRs. Axon diameter estimates with the extra-axonal diffusion time dependency are more accurate than the ones obtained using the tortuosity approximation, and the residual bias disappears with higher SNR. In contrast, axon diameter estimates without the extra-axonal diffusion time dependency consistently overestimate the true value. In Fig. 4, the percentage of intra-axonal and extra-axonal signal is reported in the range 0–10% (to help visualisation; the remaining percentage is extra-axonal signal). As expected, smaller diffusivities and larger diameters generate larger signal attenuations (5% in Fig. 4b) and are thus easier to measure. The most challenging configuration is the one reported in Fig. 3e, which is characterised by high free diffusivity and small axonal diameter.

### *In vivo* data: axonal density

**Fig. 5:**
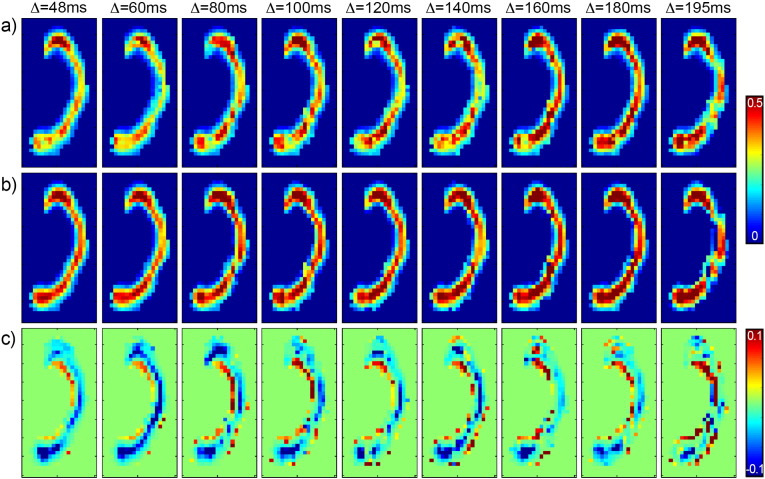
Axonal density maps at varying diffusion times. In a), the results are obtained without using the tortuosity model, while in b) the tortuosity model is used. In c), the difference between the two is shown.

Fig. 5 shows the axonal density maps in the corpus callosum at varying diffusion times, for one representative subject. The results in the upper line are obtained without using the tortuosity model (Fig. 5a), while the results in the lower line are obtained using the tortuosity model (Fig. 5b). Fig. 5c is the difference between the two. A 2-way ANOVA was performed on the two sets of maps separately, showing that the effect of diffusion time is significant (*p* < 0.05) only when the extra-axonal orthogonal diffusion is constrained by the tortuosity model. Otherwise, the diffusion time does not have a significant effect on the axonal density estimated. The location along the corpus callosum, as expected, has a significant effect (*p* < 0.05).

**Fig. 6:**
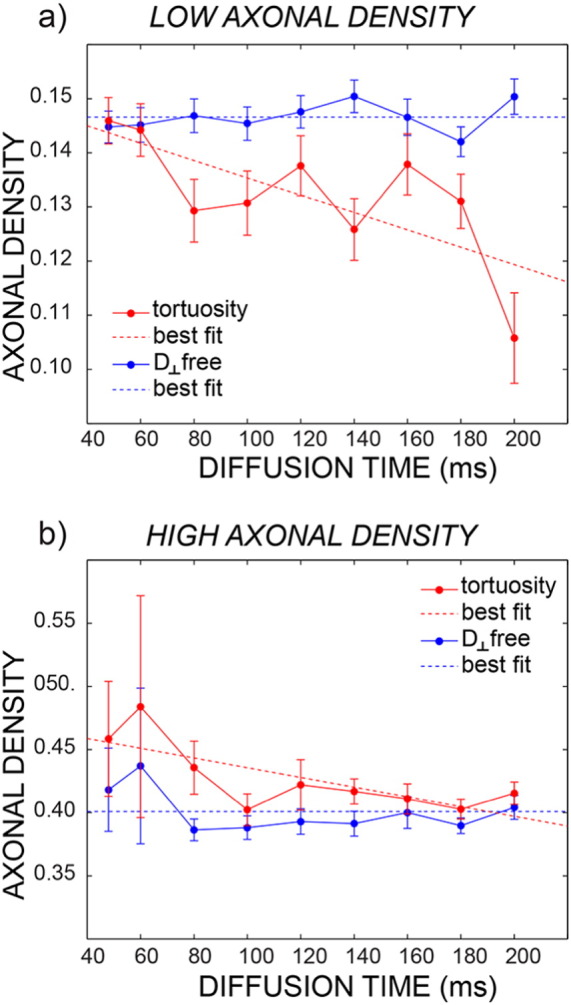
Mean axonal density and standard error at varying diffusion times for the same subject of Fig. 5. The plots are reported separately for low axonal density (a) and high axonal density (b). The blue fit is obtained without using the tortuosity model, while the red fit is obtained using the tortuosity model. Dashed lines represent the best fit of axonal density estimates over all diffusion times according to the Bayesian information criterion (see text for details).

To compare the in vivo results with the simulations, the voxels in the corpus callosum were cast into two groups, characterised by low (0.4) and high (0.4) axonal density respectively. For each diffusion time, the mean value and the standard deviation of the axonal density were calculated and reported in Fig. 6. The trend measured on real data is very similar to that predicted by simulations: while the trend for increasing diffusion time is mostly flat when the extra-axonal tensor is not constrained to follow the tortuosity model, there are strong dependencies on diffusion time when the tortuosity model is used, with a trend similar to that reported in Fig. 2b and d.

### *In vivo* data: axonal diameter

**Fig. 7:**
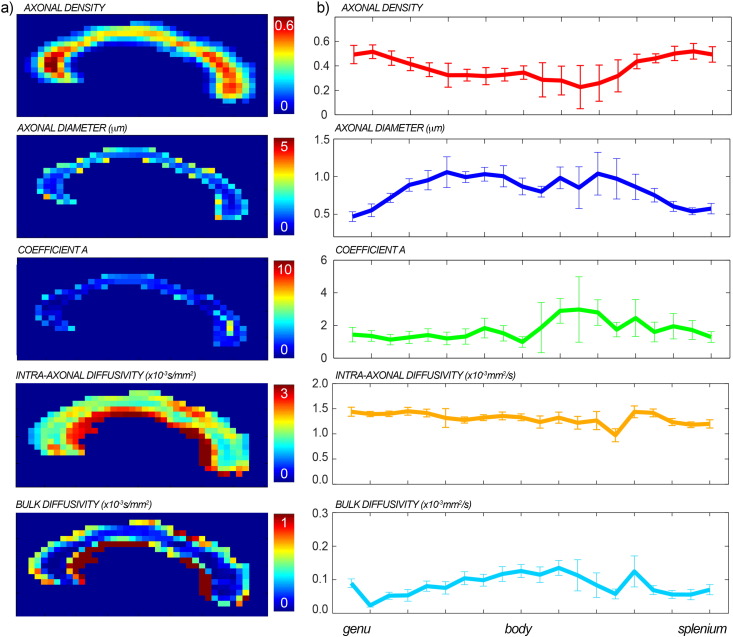
Maps of axonal density, axonal diameter, characteristic coefficient A, intra-axonal diffusivity and bulk diffusivity for one representative subject (a). In panel b, the mean profiles along the corpus callosum and the associated standard deviations over all the subjects are reported for the same parameters.

The maps of the fitted axonal density, axonal diameter, characteristic time, intra-axonal diffusivity *D*_∥_ and bulk diffusivity *D*_∞_ with the full proposed model are reported for one representative subject in Fig. 7a. Mean and standard deviations of all fitted parameters across subjects are reported in Table 1. In Fig. 7b, the mean profiles and associated standard deviations over all subjects are reported. The axonal density shows the expected high-low-high trend, while the axonal diameter has the inverse low-high-low trend. The range of the axonal diameter is between 0.5 and 1.5 μm. The characteristic coefficient shows a trend similar to that of the axonal diameter, characterised by larger values in the body as compared to genu and splenium. *D*_∥_ and *D*_∞_ have a constant trend along the corpus callosum and low standard deviations. The magnitude of *D*_∞_ is always around 10% of *D*_∥_.

**Fig. 8:**
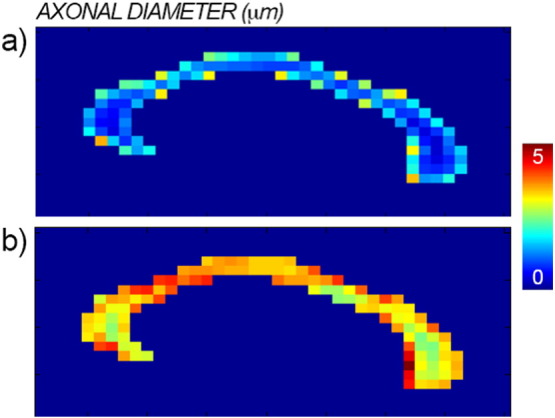
Maps of axonal diameter with (panel a) and without (panel b) accounting for the dependency on the diffusion time in the extra-axonal compartment.

Fig. 8 shows the comparison between the axonal diameter map obtained with the proposed method (panel a) and the map obtained without accounting for the diffusion time dependency in the extra-axonal compartment (panel b) for the same subject as in Fig. 7. As predicted by simulations (Fig. 3), when a fixed extra-axonal tensor is used, i.e. without diffusion time dependency, the fit returns larger estimates of the axonal density.

### *In vivo* data: stability of the fit

**Fig. 9:**
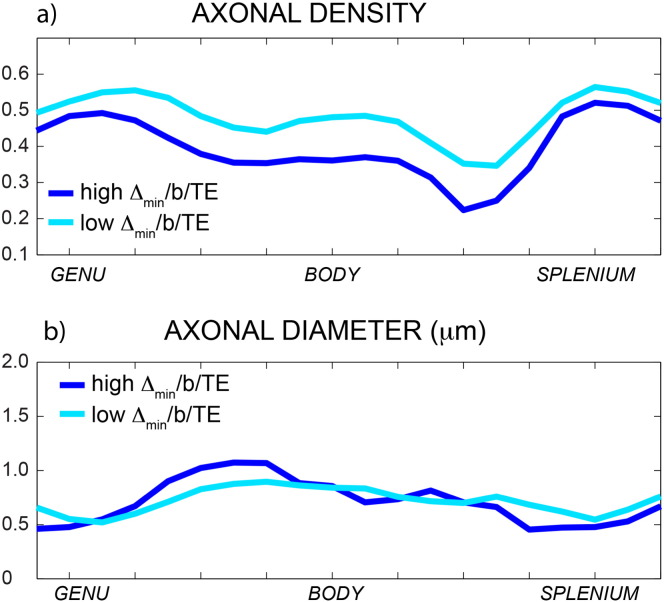
Profiles of the axonal density (a) and axonal diameter (b) along the corpus callosum. Data in dark blue are acquired with the high b-value/high Δ/high TE acquisition scheme, while data in light blue are acquired with the low b-value/low Δ/low TE acquisition scheme.

To investigate the impact of the choice of the minimum Δ on the estimated axonal density and diameter, we repeated the acquisition with two different experimental setups for one representative subject, as detailed in the Methods section. Fig. 9 shows the profiles for the axonal density and diameter. The profiles are very similar for the axonal diameter, while there is a constant bias between the two profiles of axonal density, with the second acquisition protocol having higher estimated axonal density. This is likely to be due to the difference in the echo time, and thus *T*_2_ weighting between the two acquisitions.

### *In vivo* data: correlations

**Fig. 10:**
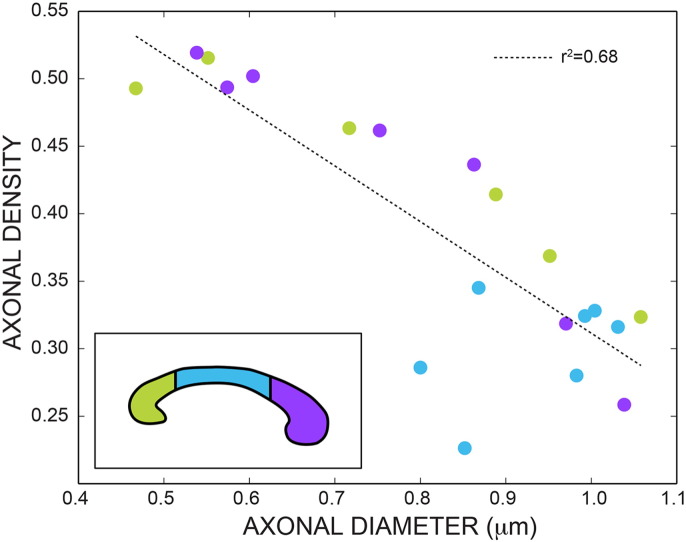
Scatterplot between axonal diameter and axonal density. The dots are colored according to the location in the corpus callosum (schematically shown in the insert). The dotted line is the linear regression (*r*^2^ = 0.68).

Fig. 10 shows the scatterplot between the axonal diameter and the axonal density estimated with the proposed model including diffusion time dependency in the extra-axonal compartment. The dots are colored according to the location in the corpus callosum. There is a clear trend, with lower diameters being associated with higher densities and vice-versa. A linear regression (dotted line) returns *r*^2^ = 0.68. As expected, the body of the corpus callosum is characterised by lower density and higher diameter, while the genu is characterised by higher density and lower diameter. The splenium has an intermediate trend between the two.

## Discussion

In this work we modify current microstructure models for diffusion imaging to estimate axonal density and diameter to correctly account for the diffusion in the extra-axonal space. We show that this strongly affects both axonal density and axonal diameter estimates, improving accuracy in ground truth simulations. We obtain human axonal diameter maps in vivo in the corpus callosum that are in close agreement with earlier histological findings.

Until a few years ago, characterising axonal properties was impossible in vivo, and thus only animal or post-mortem studies were available. With the advent of advanced diffusion methods based on multi-compartment decomposition of the signal, axonal density (Assaf et al., 2004, Alexander, 2008, Zhang et al., 2012) and diameter (Assaf et al., 2008, Alexander et al., 2010) have become accessible in the living brain. Several studies have already been published, demonstrating the potential that these techniques have. For example, the axon diameter was significantly correlated with the nerve conduction velocity (Horowitz et al., 2014), reporting *in vivo* a link between brain's structure and function. Apparent changes in axonal density were observed as a consequence of a short learning task of only two hours (Tavor et al., 2013), opening new important questions about neuroplasticity. Axonal density was reduced in focal cortical dysplasia patients (Winston et al., 2014) and in adolescents with autism spectrum disorders (Lazar et al., 2014) compared to control groups, while axonal density was reported to be a better predictor of myelin content than conventionally used FA (De Santis et al., 2014). The diffusion kurtosis model (DKI) (Jensen et al., 2005) has also been used to estimate the axonal density (Fieremans et al., 2011). DKI showed increased sensitivity in detecting white matter abnormality in schizophrenia (Zhu et al., 2015) and provided better discrimination between amnestic mild cognitive impairment from Alzheimer's (Fieremans et al., 2013), when compared to conventional DT-MRI metrics.

Despite their success, these methods are still under active development. For example, it is already well known that current methods for estimating axonal diameters are biased towards higher diameters (Alexander et al., 2010). Although some studies have investigated the impact of the acquisition parameters on the estimated biomarkers, recommending optimised protocols (De Santis et al., 2013), little attention has been paid to the impact of diffusion time in modeling diffusion in the extra-axonal compartment. Recently, it has been shown both theoretically (Novikov et al., 2014) and experimentally (Burcaw et al., 2013) that the randomness in fiber arrangement in a bundle crucially affects diffusion in the extra-axonal space, making the diffusion orthogonal to the bundle dependent on the diffusion time. In the same paper, the authors question the feasibility of axonal diameter estimation *in vivo* in humans on current clinical MRI systems as a whole, due to insufficient attenuation of the signal arising from axonal diameter differences. In this work, we modify current models for microstructural imaging to account for the extra-axonal diffusion time dependency. We show that correctly accounting for diffusion time is important for estimating axonal density, and crucial for estimating axonal diameters. We also study the feasibility of axonal diameter estimation in vivo, showing that the sensitivity crucially depends on microscopic parameters of the human tissue like the axonal diameter distribution and the true water diffusivity in the intra-axonal space.

Potential sources of bias of the tortuosity model have been discussed in recent literature (Jelescu et al., 2015). Here, we demonstrate that when the diffusion orthogonal to the main fiber orientation in the extra-axonal space is modeled using the tortuosity approximation, a bias is found in the estimated axonal density that changes with diffusion times. This result was obtained using simulations (Fig. 2) and *in vivo* (Fig. 6). This bias can be either positive or negative, depending on the axonal morphology. Notably this bias, although present, is not necessarily detrimental when all compared measures have the same diffusion time, but it becomes crucial when comparing multi-center data, or data acquired with different protocols. The proposed multi-compartment model, incorporating diffusion time dependence in the extra-axonal compartment, yields much more accurate axonal density estimates under a wide range of acquired diffusion times.

The proposed framework also solves some fundamental issues that have hampered applications of the current techniques to estimate axonal diameters, providing reproducible estimates that are in agreement with histological findings. Specifically, reconstructing axonal diameter using the methods published in the literature (Assaf et al., 2008, Alexander et al., 2010), i.e., without accounting for the dependency of the extra-axonal tensor on the diffusion time, returns values for the mean diameter in the range 5 − 10 μm. Histological measures on the human corpus callosum show that the expected range for axonal diameter is instead 0.5 − 2 μm (Aboitiz et al., 1992). By accounting for the diffusion times in the extra-axonal compartment, the diameter estimates become comparable with histology, as predicted using Monte Carlo simulations (as reported in Fig. 3) and measured in vivo (as reported in Fig. 7). We speculate that this may be one of the primary reasons for the high axonal diameter values reported in recent diffusion microstructure literature (Alexander et al., 2010). Fig. 7 also shows that the characteristic coefficient *A* has a similar profile along the corpus callosum to that reported for the axonal diameter, supporting the relationship between the two parameters reported in theory (Burcaw et al., 2015).

Another aim of the present work was also to add new information in the ongoing debate about the feasibility of axonal diameter mapping in vivo. It has been recently proposed (Burcaw et al., 2015) that clinical systems are almost completely insensitive to differences in axon diameter seen in human white matter, requiring an ability to detect differences in signal attenuation that are of the order of 3 ∗ 10^− 5^ in human white matter. It should first be noted that, were it comes to the precision of an axonal diameter estimate, small signal attenuations *per se* are not necessarily detrimental. They can be counter-balanced, up to a point, by a high SNR and a large number of measurements N. This can be formalised by considering the Cramer-Rao lower bound (CLRB) on the variance of the axonal diameter estimate (e.g., see (Alexander, 2008)). Desirable low variance (high precision) of the estimate is negatively affected by small signal changes, but positively affected by high SNR and high N. Here N generally contributes linearly to the improvement of the CLRB (i.e. CLRB ~ *N*^− 1^), which means the standard deviation of the axonal diameter estimate improves with the square root of N. In the case of the 216 measurements performed here, this achieves an improvement in precision of a factor of almost 15. However, this still puts a requirement on the magnitude of signal changes related to axonal diameters. The intra-axonal signal attenuation mostly depends on three factors: the gradient amplitude/timings, the expected axonal caliber and the intra-axonal diffusivity (see Eq. (3)). For regular clinical setups (with a maximum gradient amplitude of 40 mT/m) the resulting attenuation is often too low to be detected irrespectively of the tissue exact characteristics. For the new generation of clinical scanners, featuring maximum gradient amplitude closer to 100 mT/m, like the one used in this study, and bespoke human scanners with even stronger gradients (e.g., 300 mT/m (McNab et al., 2013) ), the attenuation varies between situations as can be seen from the strongly increasing attenuations with higher b-values up to 4000 mm^2^/s. In the worst-case scenario, i.e., small axonal diameter and fast self-diffusion coefficient, the resulting signal attenuation due to intra-axonal water is very small (0.2%), while for the best-case scenario amongst those tested the attenuation is around 5%. The effective feasibility of axonal diameter mapping now depends on the expected axonal caliber and the intra-axonal diffusivity, as shown in Fig. 4. This scenario is complicated by the fact that these two parameters have a range of true or plausible values according to the literature. Larger diameters generate larger signal attenuation, as seen comparing the first and the second columns of each row. The values used for the calculations in Fig. 4 are the smallest and largest diameters measured by histology in the human corpus callosum (Aboitiz et al., 1992). Histology is used as a gold standard but is a technique with its own shortcomings, especially because the tissue needs to be fixed, leading to tissue shrinkage (Horowitz et al., 2015). Even slightly larger axonal diameters would increase the sensitivity of the diffusion signal to axonal diameter. Furthermore, the true value of the intra-axonal diffusivity plays a role in defining the amount of signal attenuation in the intra-axonal space. It seems likely that, with the presence of microtubules, neurofilaments, organelles and macromolecules, intra-axonal diffusivity is considerably below that of free water. However, to the best of our knowledge, the only experiment that measured this quantity reported water diffusivity in pure axoplasm as 70–80% of that in pure water, as measured on the squid giant axon at 20 °C (Beaulieu, 2002), suggesting values in the range 2 − 2.4 ∗ 10^− 3^mm^2^/s at body temperature (Hasegawa et al., 1994). No measures are available for the human brain, while fitted values from CHARMED analysis are around 1.5 ∗ 10^− 3^mm^2^/s (Table 1). The lower the intra-axonal diffusivity, the larger the signal attenuation in the intra-axonal space, as shown across rows in Fig. 3. It has to be noted that the axonal diameter was correctly estimated for all the combinations of axonal diameters/diffusivities up to *D*_0_ = 1.5 ∗ 10^− 3^ mm^2^/s, and the impact of combinations generating low signal attenuation was to bias the absolute value towards larger diameters. To summarize, rather than taking a unqualified side in the ongoing discussion about feasibility or unfeasibility of axonal diameter measurements with MRI, we aimed at providing details about the conditions that need to be met to make the approach feasible, although some of them depend on parameter values that cannot be currently assumed with very high confidence.

The axonal diameter estimate obtained using the proposed framework is robust with respect to the choice of experimental parameters like the minimum sampled diffusion time and the echo time (Fig. 9). Notably, this does not apply to the axonal density, where a large impact of echo time TE is instead observed (Fig. 9). This is likely to be due to differences in T2 relaxation times between the extra- and intra-axonal water pools, and is the focus of future work. The results on axonal diameter estimates also support the idea that the contrast at the long diffusion time limit may be more informative than the contrast measured at short diffusion times, as recently proposed elsewhere (Li et al., 2014, Drobnjak et al., 2015).

We also investigated the correlation between the axonal density and the axonal diameter. As reported by histological studies, there is a clear trend where higher density is always associated with lower diameter, and vice-versa. The data reported in Fig. 10 support a linear relationship between the two biomarkers. Investigating the relationship between these two parameters is of key importance, as one of them (the axonal density) can be accessed experimentally more easily on clinical scanners, while the other (the axonal diameter) needs a more demanding acquisition protocol and high gradient strength that might not be available on current clinical setups.

### Limitations

Potential limitations of this work include a number of factors and physical effects that have not been taken into account in the modeling of multicompartment diffusion in the intra- and extra-axonal space. Exchange between the two compartments may bias the estimated signal fraction (Fieremans et al., 2010), although an agreement about the true permeability value of typical axonal membranes has not been reached yet (e.g., see (Laett et al., 2009) and (Quirk et al., 2003)), making it difficult to conclude whether exchange can be neglected or not. Furthermore, macroscopic (Zhang et al., 2012) and microscopic (Nilsson et al., 2012) orientation dispersion is expected to affect axonal diameter measurements. In this paper, we carefully selected the central section of the corpus callosum, to minimise the effect of fiber dispersion, but more complex geometry must be accounted for when extending the method to whole-brain analysis. The Poisson distribution, used as a model for axonal diameter distributions might have limited representational capacity for very narrow or fat-tailed distributions. However, no such extremes seem to occur in data published for the corpus callosum (Aboitiz et al., 1992), implying the Poisson distribution is a good parsimonious model choice. *T*_1_ differences between the extra and intra-axonal compartment might also affect the estimation of the signal fraction, although this difference, if present, is believed to be relatively small, so that many microstructural models assign the same *T*_1_ to both compartments (Deoni et al., 2008). Lastly, we fitted a single parameter for all the “unrestricted” diffusivities *D*_*r* , ∥_, *D*_*h* , ∥_ and *D*_*f*_. Although the comparative values of these diffusivities is currently being investigated (Novikov et al., 2015, Jelescu et al., 2015, Jelescu et al., 2016), there is no clear consensus in literature about it, and more focused studies are needed.

A further possible improvement of the technique is exploring a better way of fitting the signal decay equation, which contains many parameters with non-trivial dependencies. For example, the standard deviation of the parameter *A* is quite high both in simulations and in vivo. This might also limit the accuracy on the other fitted parameters. Exploring the performance of different optimization algorithms and fixing or initializing of parameters to values from fits of simpler models may prove advantageous.

## Conclusions

In this paper, we show that correctly accounting for the dependency of extra-axonal diffusion weighted signal on diffusion time strongly affects both axonal density and axonal diameter estimates. Concerning axonal density estimates, both upward and downward bias in different situations are removed by modeling extra-axonal time-dependence, showing increased accuracy in these estimates. Concerning axonal diameter estimates, we report increased accuracy in ground truth simulations and axonal diameter estimates decreased away from high values given by earlier models and towards known values in the human corpus callosum when modeling extra-axonal time-dependence. We note that relative values in earlier estimates may still be interpretable. We also stress that the signal attenuation tied to axonal diameter differences crucially depends on two parameters, the intra-axonal diffusivity and the axonal diameters, both of which have a range of true or plausible values according to the literature. In this light, we aimed at providing details about the conditions that need to be met to make axonal diameter estimates feasible, although some of them depend on parameter values (e.g. intra-axonal diffusivity) that cannot be currently assumed with very high confidence. This adds important information to the ongoing debate about feasibility of in vivo axonal diameter measurements in both advanced and clinical settings.

## Figures and Tables

**Table 1:** Mean and standard deviation of all fitted parameters for the simulations (comprising 4 axonal geometries with axonal diameter 1.26, 1.32, 1.52 and 1.94 μm, for *D*_*f*_ = 1.5 ∗ 10^− 3^ mm^2^/s) and for real data. Standard deviations are calculated across different repetitions for simulated data, and across subjects for real data. The diffusivities are expressed in 10^− 3^mm^2^/s, *f*_*r*_ is dimensionless, *A* is expressed in mm^2^ and *AD* in μm.

	Sim 1		Sim 2		Sim 3		Sim 4		In vivo	
	*Mean*	*St dev*	*Mean*	*St dev*	*Mean*	*St dev*	*Mean*	*St dev*	*Mean*	*St dev*
*D*_∥_	1.44	0.02	1.43	0.02	1.42	0.02	1.44	0.02	1.47	0.05
*D*_∞_	0.68	0.05	0.51	0.06	0.61	0.04	0.64	0.07	0.32	0.22
*f*_*r*_	0.63	0.02	0.75	0.02	0.69	0.01	0.67	0.02	0.39	0.01
*A*	1.07	0.27	1.26	0.51	1.12	0.49	1.17	0.31	0.86	0.52
*AD*	1.76	0.44	1.56	0.60	1.92	0.58	2.22	0.40	1.05	0.18

## Appendix A Supplementary data

Fig. 1S Example of signal fit in one selected voxel for Monte Carlo simulated data (left) and real data (right). Data are colored according to the orientation of the applied gradient (red along z and blue along x).

## Footnotes

1 Strictly speaking the axonal density is the intra-axonal signal (or water) fraction (dimensionless) divided by the axonal area (proportional to the squared axonal radius). However, we will use the term axonal density here to refer to the intra-axonal signal fraction alone, following the diffusion microstructure MRI literature.
