# Supplementary material for: Including diffusion time dependence in the extra-axonal space improves in vivo estimates of axonal diameter and density in human white matter
Source: Neuroimage. 2016 Apr 15;130:91–103. doi: 10.1016/j.neuroimage.2016.01.047 (PMC4819719; doi:10.1016/j.neuroimage.2016.01.047)
Supplement: Fig. 1S — Example of signal fit in one selected voxel for Monte Carlo simulated data (left) and real data (right). Data are colored according to the orientation of the applied gradient (red along z and blue along x). [file mmc1.docx]

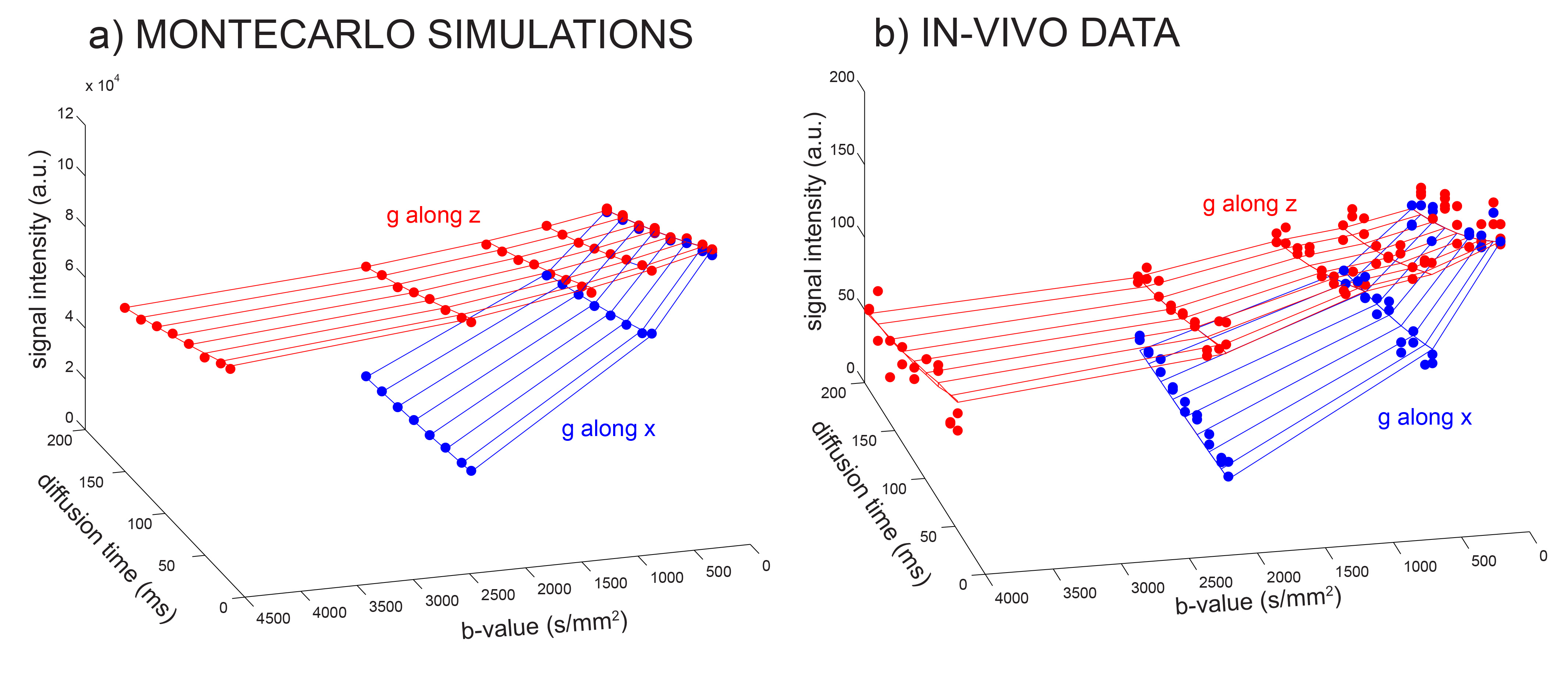


**Figure 1S**

Example of signal fit in one selected voxel for Monte Carlo simulated data (left) and real data (right). Data are colored according to the orientation of the applied gradient (red along z and blue along x).
